# Hard and crack resistant carbon supersaturated refractory nanostructured multicomponent coatings

**DOI:** 10.1038/s41598-018-32932-y

**Published:** 2018-09-28

**Authors:** S. Fritze, P. Malinovskis, L. Riekehr, L. von Fieandt, E. Lewin, U. Jansson

**Affiliations:** Department of Chemistry-Ångström, Uppsala University, SE-751 21, Uppsala, Sweden

## Abstract

The combination of ceramic hardness with high crack resistance is a major challenge in the design of protective thin films. High entropy alloys have shown in earlier studies promising mechanical properties with a potential use as thin film materials. In this study, we show that small amounts of carbon in magnetron-sputtered multicomponent CrNbTaTiW films can lead to a significant increase in hardness. The film properties were strongly dependent on the metal composition and the most promising results were observed for TaW-rich films. They crystallised in a bcc structure with a strong (110) texture and coherent grain boundaries. It was possible to deposit films with 8 at.% C in a supersaturated solid-solution into the bcc structure without carbide formation. A major effect of carbon was a significant grain refinement, reducing the column diameter from approximately 35 to 10 nm. This resulted in an increase in hardness from 14.7 to 19.1 GPa while the reduced E-modulus stayed constant at 322 GPa. The carbon-containing films exhibited extremely little plastic deformation around the indent and no cracks were observed. These results show that supersaturation of carbon into high entropy films can be a promising concept to combine superior hardness with high crack resistance.

## Introduction

In 2004 the concept of alloying several principal elements was introduced simultaneously by Yeh *et al*.^1^ as high entropy alloys (HEAs) and by Cantor *et al*.^2^ as multicomponent alloys (MCAs). In the original HEA concept, five or more elements are mixed with close to equimolar concentrations. For such an alloy a mixture of intermetallic phases is intuitively expected but instead single-phase solid solutions with simple ccp (cubic closed packed) or bcc (body centred cubic) structures are frequently observed^3^. Many new HEAs with bcc structures are based on refractory transition metals in the groups four to six such as Ti, Nb, Ta, Mo and W. An excellent example is MoNbTaW, where the phase stability and ordering of this alloy have been investigated theoretically in several studies^4–6^. Zou *et al*. have also reported superior mechanical properties for nanocrystalline MoNbTaW thin films. The latter exhibit extraordinarily high yield strengths of ~10 GPa which constitute one of the highest reported values for an alloy^7–11^. The high ductility and strength was explained by a grain refinement effect where the highest strength was observed for highly textured films with columnar grains of about 70 nm in width^7^. The mechanical properties can also be tuned by varying concentrations within the composition window. For example, the ductility of a HfTa_x_TiZr alloy was found to be significantly improved when the Ta content was reduced from 25 at.% to a concentration below 10 at.%^12^.

The aim of this study is to identify design routes to deposit harder and more crack resistant materials by reducing the grain size in HEA films following the concepts in refs^6,9^. We have used two approaches: (i) addition of small amounts of carbon and (ii) variations in metal content. The first approach is based on the observation that p-elements such as carbon can lead to grain refinement in magnetron sputtered films^13^. This is due to the limited solubility of these elements in bcc alloys. During film growth, this will lead to an enrichment of the p-elements on the surface of the growing grains and at a critical point, a renucleation of new grains giving a more-fine grained microstructure. The second approach is based on the assumption, that also the metals in the alloys can segregate to the surface leading to a similar renucleation step as observed for the p-elements. For example, we have recently demonstrated that Cr is segregated to the grain boundaries in carbidic CrNbTaTiWC films^14^. The driving force for this, is partly a reduction in lattice distortion when the smaller Cr atoms are removed from the bulk of the grains.

In this study, we have investigated the influence of carbon addition and metal content on the mechanical properties of magnetron-sputtered CrNbTaTiW films. We have deposited two sets of films: one without carbon and a second with about 8 at.% C. Within each set, we have deposited films with a near-equimolar composition, a Nb-rich film and finally a Ta/W-rich film. The phase composition and microstructure of all films were studied with X-ray diffraction (XRD), X-ray photoelectron spectroscopy (XPS) and electron microscopy (SEM and TEM). Finally, hardness and ductility measurements were performed to investigate the mechanical properties.

## Results

A series of CrNbTaTiW films with different relative metal concentrations were deposited at 300 °C. The Nb-rich films were found to be less dense and hard (<5.2 GPa) and they were therefore excluded from further investigations. In contrast, Ta/W-rich films with the composition Cr_3_Nb_12_Ta_41_Ti_3_W_41_ (labelled TaW) and a near-equimolar film (labelled NE) with the composition Cr_29_Nb_16_Ta_16_Ti_21_W_18_ were found to exhibit more interesting mechanical properties and they were therefore selected for detailed investigations. To study the influence of carbon, two films labelled NE(C) and TaW(C) were deposited with about 8 at.% C. In addition, two carbidic films named TaWC and NEC from an earlier study on carbides from ref.^14^ were also included in the study to enable comparisons with more carbon-rich films. For all the carbon-containing films, the magnetron settings for the metal targets were kept constant to obtain the same relative concentration of the metals as in the TaW and NE samples. However, the characteristics of the sputter process resulted in a change in the Ti/Cr ratio for the metallic to the carbidic near-equimolar samples. The composition, structure and mechanical properties of the selected films are summarised in Table 1.

**Table 1:** Sample name, chemical composition from ERDA and TEM EDS, structure from XRD, hardness (H), reduced elastic modulus (Er), and H/Er ratio from nanoindentation.

Sample	Metal content [at.%]					C [at.%]	Structure	H [GPa]	E_r_ [GPa]	H/E_r_
	Cr	Nb	Ta	Ti	W					
TaW	3	12	41	3	41	0	A2 (bcc)	14.7 ± 0.3	321 ± 5	0.046
TaW(C)	4	12	40	4	40	8	A2 (bcc)	19.1 ± 0.3	322 ± 3	0.059
TaWC	6	10	39	6	39	38	B1 (NaCl type)	26.8 ± 2	390 ± 34	0.069
NE	26	19	16	21	18	0	A2 (bcc)	13.3 ± 0.3	185 ± 3	0.072
NE(C)	29	17	16	17	21	8	amorphous	14.9 ± 0.4	210 ± 5	0.071
NEC	21	13	22	22	22	40	B1 (NaCl)	15.7 ± 2	287 ± 34	0.055

**Figure 1:**
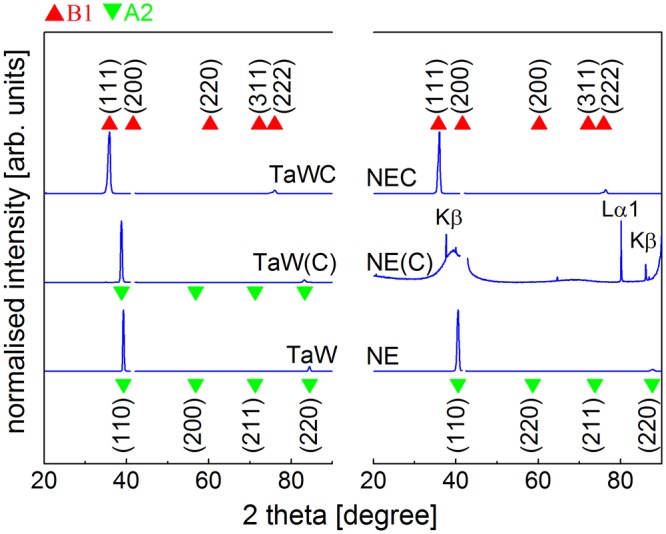
θ-2θ XRD scans (normalised with respect to the highest intensity) of the carbon-free TaW and NE films (bottom),the TaW(C) and NE(C) film with about 8 at.% C (middle) and the carbidic TaWC and NEC films (top). The sapphire (006) reflection at 2θ = 41.68° was removed prior to the normalisation. The strongly textured films show only (110) and (220) peaks for the A2 films (green triangles) and (111) and (222) peaks from the carbide films (red triangles).

Figure 1 shows the normalised θ-2θ X-ray diffractograms of the films in Table 1. The diffraction patterns of the TaW and the NE films only show (hh0) peaks from a cubic A2 (bcc, Im $\bar{3}$ m) phase with a <110> preferred orientation. The unit cell parameters of the TaW and the NE films were determined to a = 3.24 Å and a = 3.15 Å respectively. No indications of additional phases were found in the diffraction patterns. The addition of 8 at.% C to the TaW film led to an expansion of the unit cell to a = 3.28 Å while retaining the A2 structure. No carbide peaks were observed in the diffractogram from the TaW(C) film. However, a minor increase in the FWHM of the (110) peak from 0.31° to 0.41° was observed. In contrast, the addition of carbon to the NE sample resulted in an amorphous film and the diffraction pattern exhibited only Lα1 and Kβ lines of the Al_2_O_3_ (006) and (0012) peaks. Finally, the carbon rich films from ref.^14^ exhibited a cubic NaCl-type (Fm $\bar{3}$ m) structure with a preferred <111> orientation.

High resolution XPS spectra were acquired from all films (see Supplementary Fig. S1). The presence of carbon in the film leads to a shift of the metal core level towards higher binding energies compared to the two metallic films. The C1s spectra showed a broad peak at about 281.5 eV, which can be attributed to C-Me bonding. The amorphous NE(C) and the carbidic NEC and TaWC films also showed a small and broad peak at 285.5 eV corresponding to about 10–20% of the total C1s intensity, which can be attributed to free carbon (C-C).

The mechanical properties (i.e. hardness and reduced elastic modulus) of the films are summarised in Table 1. The TaW film showed a hardness of 14.7 ± 0.3 GPa and a reduced elastic modulus of 321 ± 5 GPa. The carbon-containing TaW(C) exhibited a significantly higher hardness of 19.1 ± 0.3 GPa and a reduced elastic modulus of 322 ± 3 GPa. A further hardness increase to 26.8 ± 2.2 GPa and reduced elastic modulus to 390 ± 34 GPa was observed for the carbidic TaWC film. A different behaviour is observed for the near-equimolar series. The hardness and the reduced elastic modulus were determined to 13.3 ± 0.3 GPa and 185 ± 3 GPa respectively. The amorphous NE(C) film showed a slightly higher hardness and elastic modulus of 14.9 ± 0.4 GPa and 210 ± 5 GPa, respectively. The formation of a multicomponent carbide phase in the NEC film led to no significant improvement of the mechanical properties. The H/E_r_ ratio of all films is below 0.1, indicating a limited ductility.

**Figure 2:**
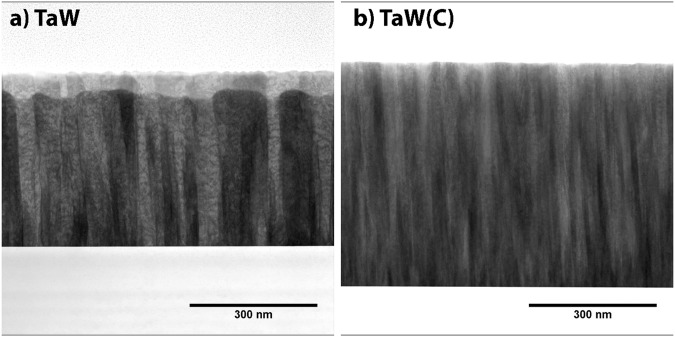
STEM bright field cross-section overview of (a) TaW (~400 nm thick) and (b) TaW(C) (~500 nm thick. The FIB lamella (and in particular the top region) of the TaW sample is thinner compared to the TaW(C) sample resulting in a contrast variation.

**Figure 3:**
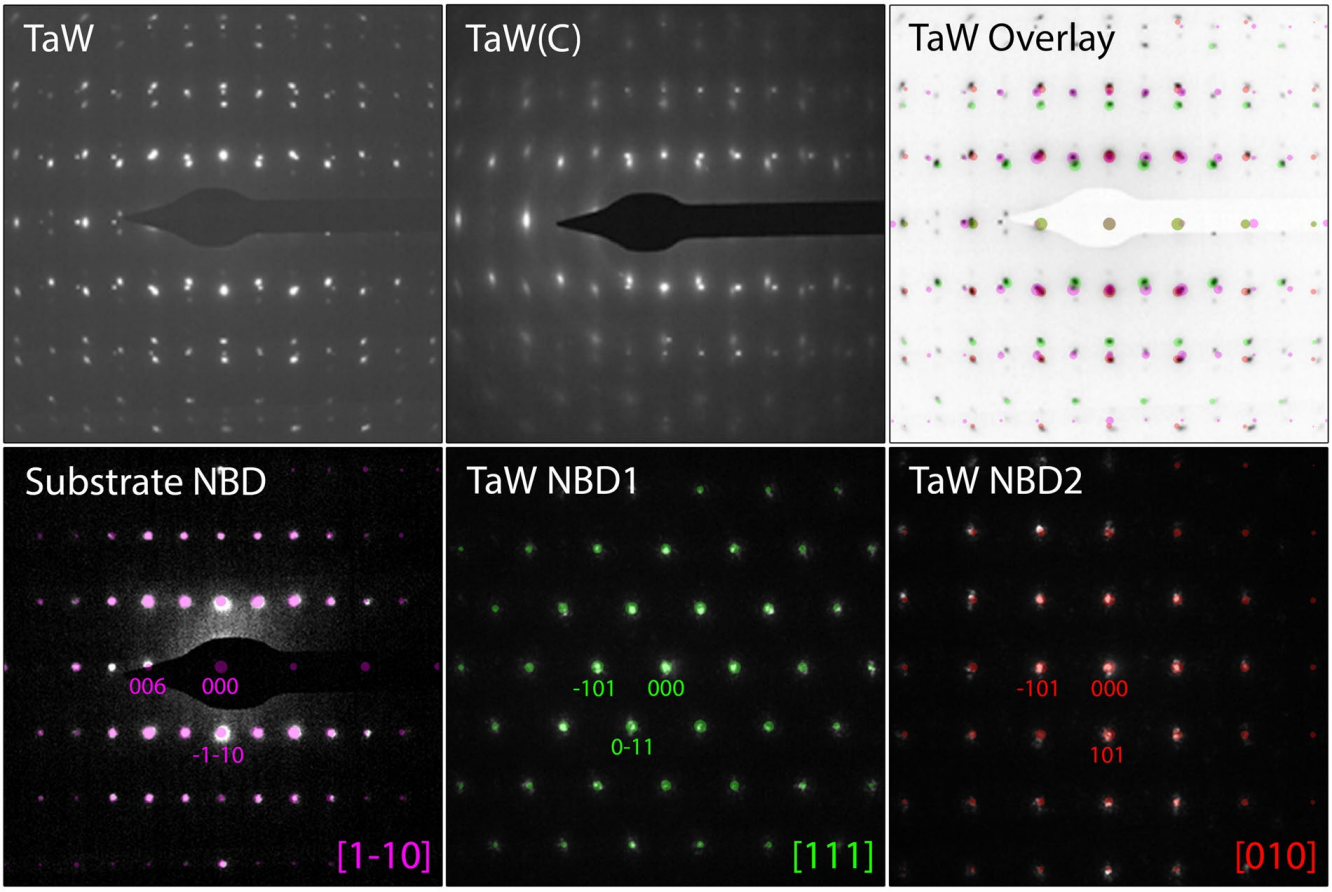
Top row shows SAED from the complete film cross-section of the TaW and TaW(C) sample. Bottom row shows nano-beam diffraction data: NBD1and NBD2 represent diffraction patterns of two individual columns. The purple marked spots correspond to the sapphire substrate. The green spots correspond to reflections from the [111] zone axis and the red spots correspond to reflections from the [010] zone axis.

The results above clearly show that the TaW-rich films exhibits the most promising mechanical properties and we have therefore studied the microstructure of these films in more detail. Bright field STEM cross-section micrographs of the TaW and TaW(C) films are presented in Fig. 2. Both films exhibited a columnar-like structure extending from the substrate to the film surface. The width of the columns in the TaW film was 35 ± 5 nm. The addition of carbon in the TaW(C) film led to a significant column refinement and the column width decreased to an average value of 10 ± 5 nm. No indication of carbide formation could be observed which is in agreement with the SAED data presented in Fig. 3.

Selected area electron diffraction (SAED) patterns recorded over the whole film cross section and nano-beam diffraction (NBD) patterns on two individual columns are presented in Fig. 3. The diffraction patterns of both films were indexed using an A2 (bcc, Im $\bar{3}$ m) structure in agreement with the XRD results in Fig. 1. The reflection spots in the TaW film appear to be sharper than for the TaW(C), which is mainly attributed to the smaller column width in the TaW(C) film. The NBD patterns of the TaW sample show that the columns have two different in-plane crystal orientations, [111] and [010], that share a common −101 reflection. A combination of these two pattern indexes all reflections in the overlay pattern. Furthermore, no reflections corresponding B1 carbide structure were observed in the SAED pattern revealing the absence of carbide precipitates.

**Figure 4:**
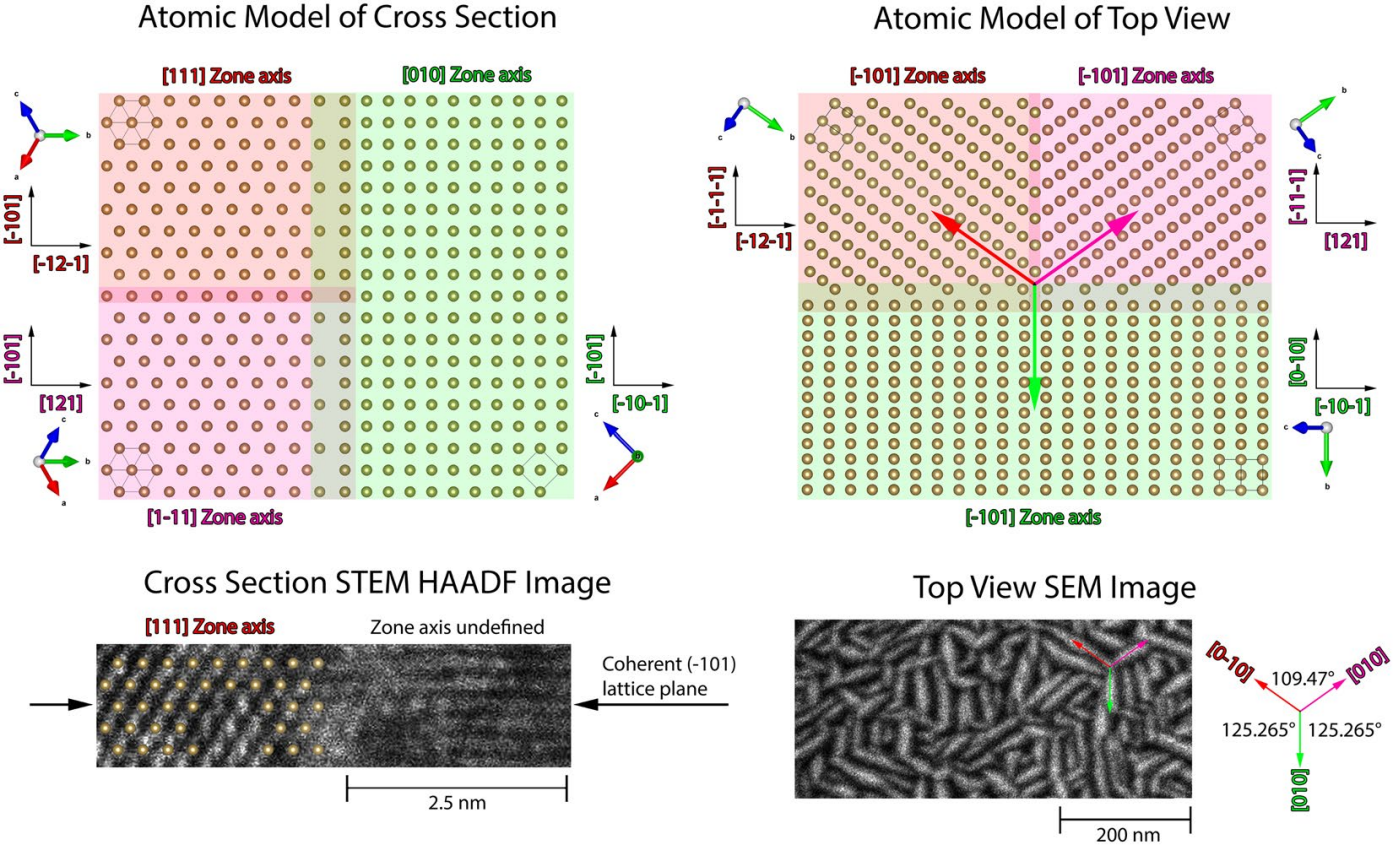
(**a**) Atomistic model of the film cross section revealed from nano-beam diffraction. (**b**) HR-STEM of one grain boundary. (**c**) Atomistic model of the film top view extrapolated from the film cross section. (**d**) SEM top view of TaW with the crystallographic orientation of three zone axes pointed out by arrows.

Figure 4(a) is the atomistic model of the present zone axes extracted from the nano-beam diffraction. Two <111> zone axes that cannot be distinguished in cross-sectional view are included in the atomic model. This was necessary to explain the growth directions identified in the top view SEM analysis in Fig. 4(d). A STEM HAADF image of a grain boundary is shown in Fig. 4(b). The zone axis of the left grain was unambiguously identified as the [111] zone axis and the atom positions are marked by the golden dots. The zone axis of the right grain could not be identified. However, nano-beam diffraction only revealed the presence of the <111> and <010> zone axes that share a common (−101) lattice plane. This is confirmed by the high resolution STEM HAADF micrograph where it can be seen that the (−101) lattice planes are coherent in both grains as suggested by the coinciding reflections in the electron diffraction patterns. The micrograph also shows that the orientation changes seamlessly at the grain boundary, even though the atomic configuration at the grain boundary cannot be resolved. Figure 4(c) depicts the atomistic top view model of the films. The model is an in-plane projection of the cross section model. Because of the strict crystallographic orientation relationship between the columns found by the electron diffraction analysis, the [010] directions of the differently oriented columns exhibit angles of either 109.47° or 125.265°. This angular relationship is found in the SEM top view image of the TaW sample depicted in Fig. 4(d) and gives further evidence for the strict orientation relationship between the columnar grains.

**Figure 5:**
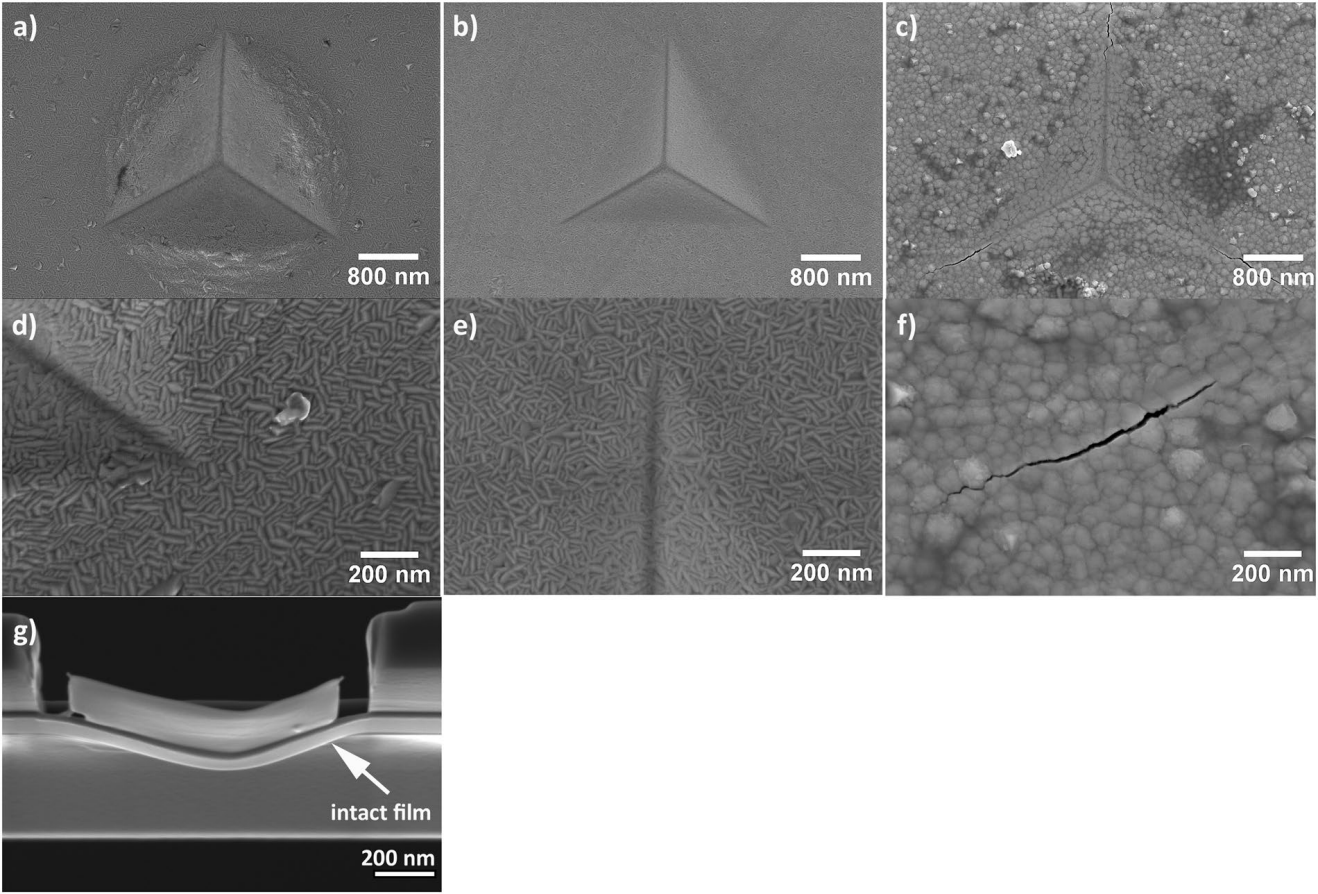
SEM top view images of (**a**) TaW, (**b**) TaW(C) and (**c**) TaWC. A close up on the indentation corners are given in (**d**) TaW, (**e**) TaW(C) and (**f**) TaWC. The indentation depth was set to 500 nm for all films. (**g**) FIB lamella prepared under the indent of the TaW(C) film.

The crack resistance of the films was tested by 500 nm deep indents into the films using a Berkovich tip. SEM top-view images of the indented surfaces of TaW, TaW(C) and TaWC films are shown in Fig. 5. The indent of the TaW film (Fig. 5(a,d)) shows no cracks but major pile-ups along the edges. In contrast, the indent into the TaW(C) showed no indication of cracks or pile-ups (see Fig. 5(b,e)). The indentation of the TaWC film resulted in severe radial cracks at the indent corners (Fig. 5(c,f)). The results show that the carbon-containing TaW(C) film exhibits a high crack resistance combined with an unusually high hardness. The high crack resistance of this film can also be seen in Fig. 5(g). Attempts to prepare TEM samples using a FIB technique, failed in our case, due to the high stresses induced by the diamond tip. This created a highly bent free-standing film without any cracks, illustrating the special mechanical properties of this material, see Fig. 5(g).

## Discussion

The results show that CrNbTaTiW high entropy alloys with an extensive solid solubility on the metal lattice can be synthesised by magnetron sputtering. Furthermore, it is also shown that a single phase solid solution HEA can be obtained in a wide compositional range. Four out of the five elements in the present study (Cr, Nb, Ta and W) crystallize in a bcc structure (A2, Im $\bar{3}$ m) and therefore an extensive solid solubility of these elements can be expected^15–17^. However, Cr is prone to form MCr_2_ (M = Nb, Ta and Ti) Laves phases and we therefore attribute the formation of a single phase HEA containing Cr and Ti to the high entropy of mixing and to kinetic constrains present during the PVD process.

W has a maximum solubility (at ~2200 °C) of 0.3 at.%^18^ and Ta of 2.7 at.% carbon^19^. The addition of 8 at.% carbon is therefore far above the expected solubility limit in a bcc alloy. The high carbon content caused an amorphisation of the NE(C) sample. This can be explained by the large atomic size difference between carbon and the metallic constituents. It is well established that an alloy consisting of three or more elements with a large atomic size mismatch (>12%) has the tendency to form a metallic glass^20^. In contrast, XRD of the TaW(C) film showed a single bcc phase without any indications of metal carbide. The expansion of the cell parameter of the bcc phase in the TaW(C) film from 3.24 Å to 3.28 Å suggests that the carbon forms a supersaturated solid solution, presumably with carbon in the octahedral sites of the structure^21–23^. This hypothesis is supported by the absence of carbidic precipitates in the TEM analysis and C-C contribution in the C1s XPS spectra. Deposition of highly supersaturated or metastable films are frequently observed in magnetron sputtering due to the high quenching rate of the impinging atoms leading to reduced diffusion distances on the surface of the growing film^24^.

The electron diffraction and the XRD results of the TaW-series suggest a highly textured or epitaxial (110) growth on the Al_2_O_3_ (001). The TaW and the TaW(C) films exhibit a typical columnar microstructure of 35 ± 5–10 ± 5 nm width, where the columns extend from the substrate to the film surface. The columns of both films follow a strict orientation relationship with densely packed grain boundaries. The nano-beam diffraction in Fig. 4 shows that the textured and columnar grains are highly coherent with a strict orientation relationship between the grains. This leads to the surface morphology shown in Fig. 4(d). This columnar microstructure differs significantly from observed microstructures of previously investigated super saturated solid solutions^25^.

The hardness of the NE and TaW films are 13.3 ± 0.3 GPa and 14.7 ± 0.3 GPa, respectively. This hardness is beyond expectation based on the linear rule of mixture of the elements. However, the highly distorted lattice in the HEAs is expected to give rise to significant solution hardening. The fact that the softer NE film has a higher lattice distortion than the harder TaW film, shows that there is no direct correlation between lattice distortion and hardness. Furthermore, several recent studies on bcc films deposited by magnetron sputtering have demonstrated very high hardness values for e.g. W^23,26^ and TaW^27^ films. Wang *et al*. have reported a hardness of 14.3 GPa for TaW thin films and explained the high hardness based on a nanocrystalline microstructure^27^. Feng *et al*.^28^ have recently investigated the size effect on nanocrystalline NbMoTaW thin films. The results showed that the minimum grain size for solid solution hardening in this alloy is around 40 nm. The hardest films however were found for a grain size of around 10 nm because of source strengthening. These reports, together with the present analysis of the films microstructure in our study, suggest that the hardness of the thin films is mainly controlled by other factors, such as the microstructure and stresses^29,30^. We suggest that the extraordinary hardness of the carbon-free TaW films in our study is a result of the highly dense packed nanocolumns and the blocking of the dislocation movement across the column boundaries.

When C is added to our alloys, the hardness increases. The hardness of the XRD amorphous NE(C) film is around 1 GPa higher than for the carbon-free NE film. Amorphous metallic glasses are known to be hard but brittle and consequently a nanocrystalline microstructure is not required *per se* to obtain a high hardness for these alloys. A significant hardness increase to 19.1 GPa, which is commonly found in ceramics, is observed for the TaW(C) compared to TaW. This film is not a carbide but should be described as a supersaturated metallic bcc alloy. The high hardness is explained by grain refinement, by which the column width decreases from 35 ± 5 nm to 10 ± 5 nm, while the dense packed column boundaries are retained. The grain refinement is in agreement with general observations in magnetron sputtering that the addition of p-elements such as B, C, N and Si in small amounts tend to form films with smaller grain sizes^31,32^.

The deformation tests show a very interesting effect of carbon on the toughness of the films^33,34^. The TaW films exhibits significant plastic deformation leading to a pile-up around the indent. The TaWC films showed cracks originating from the indent corners. This is expected for a transition metal carbide, which combines a high hardness with a more ceramic and brittle behaviour. In contrast, the carbon supersaturated film shows a completely different behaviour. For the TaW(C) no cracks or pile-ups were observed. This suggests that ceramic hardness can be combined with metallic toughness in carbon-containing bcc structures. Previous studies have shown similar results when alloying group six metals with p-block elements and it was suggests that the toughening effect is caused by the non-columnar microstructure^25,35^. However, we observe toughness in highly columnar films. We, therefore, propose that the toughness is not solely defined by the microstructure but also other effects such as the chemical composition (chemical bonding nature) of the alloy.

## Conclusions

In summary, we deposited CrNbTaTiW films with different carbon and metal concentrations by magnetron co-sputtering. XRD results show that all film except one crystallise in a cubic bcc structure. The addition of 8 at.% carbon in the TaW-rich series leads to the formation of supersaturated solid solutions with carbon in octahedral sites without any formation of carbides. This caused a grain refinement to significantly smaller grains (~10 ± 5 nm) compared to the pure metallic TaW-rich film, which led to an increase in hardness from 14.7 to 19.1 GPa. Nanoindentation experiments revealed that the TaW(C) films combine ceramic hardness with high crack resistance which is attributed to the unique microstructure (small grains with coherent grain boundaries). Our results show alloying intrinsically brittle metals (e.g. W) with carbon and forming supersaturated solid solutions have the potential to act as new tough protective thin films.

## Experimental

The films were deposited using an ultra-high vacuum DC-magnetron sputtering system (base pressure <10^−8^ Pa) equipped with four magnetrons^36^. All HEA thin films were deposited by non-reactive DC-magnetron sputtering employing the following two-inch circular targets: pre alloyed Ti/Cr (1:1), Nb, segmented Ta/W (1:1) and a C target. All targets had a claimed purity of 99.9%. The substrate holder was rotating during the deposition in order to enhance the film homogeneity. Si (001) and α-Al_2_O_3_ (001) substrates were used. Prior to film growth, the substrates were heated to 300 °C for at least 1 h to minimise the risk of temperature gradients. An Ar^+^ plasma was ignited at 0.6 Pa, using a 42 sccm Ar gas flow rate and a DC bias voltage of −100 V was applied to the substrate table during all depositions. The currents on the Nb, Ti/Cr and Ta/W targets varied while the current on the C target was either 0 or 40 mA.

Bragg-Brentano and grazing incidence (GI) (ω = 2°) XRD was performed using a Philips X’Pert MRD diffractometer with Cu Kα radiation. A parallel beam setup with a Göbel mirror on the primary side and a parallel plate collimator with a 0.27° acceptance angle on the secondary side was used. The peak positions and peak widths were determined by curve fitting of the peaks and measured as the full width at half maximum. Lattice parameters were determined using the observed (110) and (200) peaks assuming a body centred cubic structure.

The chemical composition of the samples was determined by Time-of-Flight Energy ERDA (ToF-E-ERDA) analysis, carried out at the Tandem Accelerator Laboratory at Uppsala University. For these measurements, 36 MeV ^127^I^8+^ ions were used as projectile species and the base pressure in the experimental chamber was <10^−4^ Pa. A detailed description of the detection system and the analysis software can be found in refs^37,38^.

X-ray photoelectron spectropscopy (XPS) with a PHI Quantum 2000 was used to determine the chemical bonding environment in the films. Both employ monochromatic Al Kα radiation and a 45° photoelectron take-off angle. High resolution spectra were acquired after sputter etching with 1 keV Ar^+^ ions for 10 minutes to investigate the bulk properties of the coating. The sputter-etched area was 1 × 1 mm^2^ and the analysis region was set to be 200 µm in diameter.

A Zeiss Merlin SEM microscope was used to study the coating morphology. An in-lens detector and a 5 keV acceleration voltage were used to investigate the surface morphologies of the films deposited on Al_2_O_3_ substrates. Electron transparent cross-section TEM samples were prepared from as-deposited films using a FEI Strata DB235 FIB/SEM. The TEM investigations were carried out in STEM mode with a 200 kV acceleration voltage on a probe corrected FEI Titan Themis instrument equipped with the SuperX EDS system. For µProbe STEM, the electron beam in the TEM was set up to a nearly parallel beam condition, focused to approximately 5 nm, after which a STEM image was recorded. The beam was then positioned at different areas of interest in the µProbe STEM image to acquire nano-beam diffraction (NBD) patterns with high lateral resolution. The software tools CrysTBox^39–41^ and JEMS^42^ were used to evaluate the diffraction patterns.

The mechanical properties (i.e. Young’s modulus, hardness and toughness) were determined with a CSM Instruments Ultra Nano Hardness Tester (UNHT) equipped with a Berkovich diamond tip. Load-displacement curves were recorded on 20 different spots with 75 nm displacement and loading and unloading rates of 2.5 mN/min. The hardness (H) and reduced Young’s modulus (E_r_), were evaluated from the load-displacement curves as described by Oliver and Pharr^43^. Average values based on at least 15 indentation spots were used. The toughness was tested via indents where the indentation depth was set to 500 nm.

## Electronic supplementary material

Supplementary Dataset 1

## References

[1] Yeh JW (2004). Nanostructured high-entropy alloys with multiple principal elements: Novel alloy design concepts and outcomes. Adv. Eng. Mater..

[2] Cantor B., Chang I.T.H., Knight P., Vincent A.J.B. (2004). Microstructural development in equiatomic multicomponent alloys. Materials Science and Engineering: A.

[3] Miracle DB, Senkov ON (2017). A critical review of high entropy alloys and related concepts. Acta Mater..

[4] Körmann F, Ruban AV, Sluiter MHF (2016). Long-ranged interactions in bcc NbMoTaW high-entropy alloys. Mater. Res. Lett..

[5] Körmann Fritz, Sluiter Marcel (2016). Interplay between Lattice Distortions, Vibrations and Phase Stability in NbMoTaW High Entropy Alloys. Entropy.

[6] Singh, P., Smirnov, A. V. & Johnson, D. D. Ta-Nb-Mo-W refractory high-entropy alloys: anomalous ordering behavior and its intriguing electronic origin. **055004**, 1–6 (2017).

[7] Zou Y, Ma H, Spolenak R (2015). Ultrastrong ductile and stable high-entropy alloys at small scales. Nat. Commun..

[8] Zou Yu, Wheeler Jeffrey M., Ma Huan, Okle Philipp, Spolenak Ralph (2017). Nanocrystalline High-Entropy Alloys: A New Paradigm in High-Temperature Strength and Stability. Nano Letters.

[9] Zou Y, Maiti S, Steurer W, Spolenak R (2014). Size-dependent plasticity in an Nb25Mo25Ta 25W25 refractory high-entropy alloy. Acta Mater..

[10] Zou Y (2017). Fracture properties of a refractory high-entropy alloy: *In situ* micro-cantilever and atom probe tomography studies. Scr. Mater..

[11] Zou, Y. Nanomechanical studies of high-entropy alloys. *J. Mater. Res*. 10.1557/jmr.2018.155 (2018).

[12] Huang H (2017). Phase-Transformation Ductilization of Brittle High-Entropy Alloys via Metastability Engineering. Adv. Mater..

[13] Mayrhofer PH, Mitterer C, Hultman L, Clemens H (2006). Microstructural design of hard coatings. Prog. Mater. Sci..

[14] Malinovskis P (2018). Synthesis and characterization of multicomponent (CrNbTaTiW)C films for increased hardness and corrosion resistance. Mater. Des..

[15] Guo NN (2015). Microstructure and mechanical properties of refractory MoNbHfZrTi high-entropy alloy. Mater. Des..

[16] Senkov ON, Jensen JK, Pilchak AL, Miracle DB, Fraser HL (2018). Compositional variation effects on the microstructure and properties of a refractory high-entropy superalloy AlMo0.5NbTa0.5TiZr. Mater. Des..

[17] Coury Francisco Gil, Butler Todd, Chaput Kevin, Saville Alec, Copley John, Foltz John, Mason Paul, Clarke Kester, Kaufman Michael, Clarke Amy (2018). Phase equilibria, mechanical properties and design of quaternary refractory high entropy alloys. Materials & Design.

[18] Goldschmidt J, Brand JA (1963). The tungsten-rich region of the system tungsten-carbon. J. LESS-COMMON Met..

[19] Horz, G., Lindenmaier, K. & Klaiss, R. High-temperature solid solubility limit of carbon in niobium and tantalum. **35**, 97–105 (1974).

[20] Takeuchi A, Inoue A (2005). Metallic Glasses By Atomic Size Difference, Heat of Mixing and Period of Constituent Elements and Its Application To Characterization of the Main Alloying Element. Mater. Trans..

[21] Leltmrs M, Pauleau’ Y, Gouy-Pailler P (1992). Very hard solid-solution-type tungsten-carbon coatings deposited by reactive magnetron sputtering. Mater. Lett..

[22] Gouy‐Pailler P, Pauleau Y (1993). Tungsten and tungsten–carbon thin films deposited by magnetron sputtering. J. Vac. Sci. Technol. A Vacuum, Surfaces, Film..

[23] Palmquist JP (2003). Magnetron sputtered W-C films with C60as carbon source. Thin Solid Films.

[24] Jansson U, Lewin E (2013). Sputter deposition of transition-metal carbide films - A critical review from a chemical perspective. Thin Solid Films.

[25] Greczynski G (2016). Nitrogen-doped bcc-Cr films: Combining ceramic hardness with metallic toughness and conductivity. Scr. Mater..

[26] Yang L (2017). Highly hard yet toughened bcc-W coating by doping unexpectedly low B content. Sci. Rep..

[27] Wang CL, Zhang M, Chu JP, Nieh TG (2008). Structures and nanoindentation properties of nanocrystalline and amorphous Ta-Wthin films. Scr. Mater..

[28] Feng XB (2017). Size effects on the mechanical properties of nanocrystalline NbMoTaW refractory high entropy alloy thin films. Int. J. Plast..

[29] Javaid Farhan, Durst Karsten (2018). Stress-driven grain boundary movement during nanoindentation in tungsten at room temperature. Materialia.

[30] Maier-Kiener V, Schuh B, George EP, Clemens H, Hohenwarter A (2017). Nanoindentation testing as a powerful screening tool for assessing phase stability of nanocrystalline high-entropy alloys. Mater. Des..

[31] Musil J, Vlček J (1998). Magnetron sputtering of films with controlled texture and grain size. Mater. Chem. Phys..

[32] Malinovskis P, Palisaitis J, Persson POÅ, Jansson U, Lewin E (2017). Synthesis and characterisation of Mo-B-C thin films deposited by non-reactive DC magnetron sputtering. Surf. Coatings Technol..

[33] Soler R (2018). Fracture toughness of Mo2BC thin films: Intrinsic toughness versus system toughening. Mater. Des..

[34] Gleich S (2018). Modifying the nanostructure and the mechanical properties of Mo2BC hard coatings: Influence of substrate temperature during magnetron sputtering. Mater. Des..

[35] Yang L (2018). Small atoms as reinforced agent for both hardness and toughness of Group-VIB transition metal films. J. Alloys Compd..

[36] Johansson K, Riekehr L, Fritze S, Lewin E (2018). Surface & Coatings Technology Multicomponent Hf-Nb-Ti-V-Zr nitride coatings by reactive magnetron sputter deposition. Surf. Coat. Technol..

[37] Janson, M. S. *CONTES Instruction Manua*l. (2004).

[38] Zhang Y (1999). Detection efficiency of time-of-flight energy elastic recoil detection analysis systems. Nucl. Instruments Methods Phys. Res. Sect. B Beam Interact. with Mater. Atoms.

[39] Klinger M, Polívka L, Jäger A, Tyunina M (2016). Quantitative analysis of structural inhomogeneity in nanomaterials using transmission electron microscopy. J. Appl. Crystallogr..

[40] Klinger M (2017). More features, more tools, more CrysTBox. J. Appl. Crystallogr..

[41] Klinger M, Jäger A (2015). Crystallographic Tool Box (CrysTBox): Automated tools for transmission electron microscopists and crystallographers. J. Appl. Crystallogr..

[42] Stadelmann, P. JEMS version 4.6031U2017 (2017).

[43] Oliver WCC, Pharr GMM (1992). Improved technique for determining hardness and elastic modulus using load and displacement sensing indentation experiments. J. Mater. Res..

