# Supplementary material for: Hard and crack resistant carbon supersaturated refractory nanostructured multicomponent coatings
Source: Sci Rep. 2018 Sep 28;8:14508. doi: 10.1038/s41598-018-32932-y (PMC6162281; doi:10.1038/s41598-018-32932-y)
Supplement: Supplementary file 1 — Supplementary Dataset 1 [file 41598_2018_32932_MOESM1_ESM.pdf]

Supplementary information

Hard and crack resistant carbon supersaturated refractory nanostructured multicomponent coatings

S. Fritze*, P. Malinovskis, L.Riekehr, L. von Fieandt, E. Lewin, U. Jansson

Department of Chemistry-Ångström, Uppsala University, SE-751 21 Uppsala, Sweden

*Corresponding author: e-mail stefan.fritze@kemi.uu.se, phone +46 18 4713724, fax +46 18 513548

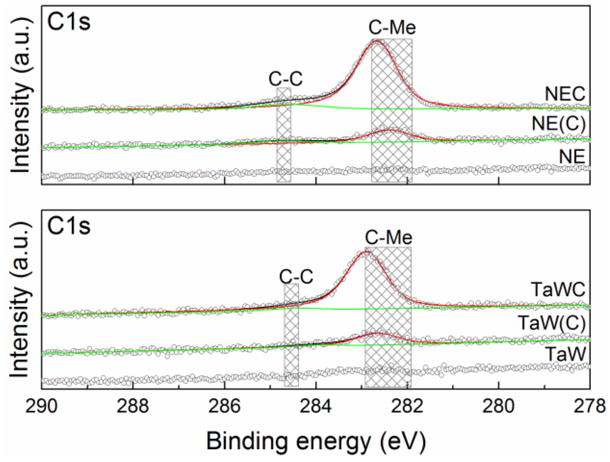

Fig. SI 1 *XPS C1s spectra of the NE, NE(C), NEC, TaW, TaW(C) and TaWC films. The literature positions of the C-C and the C-Me contributions are indicated in the spectra*

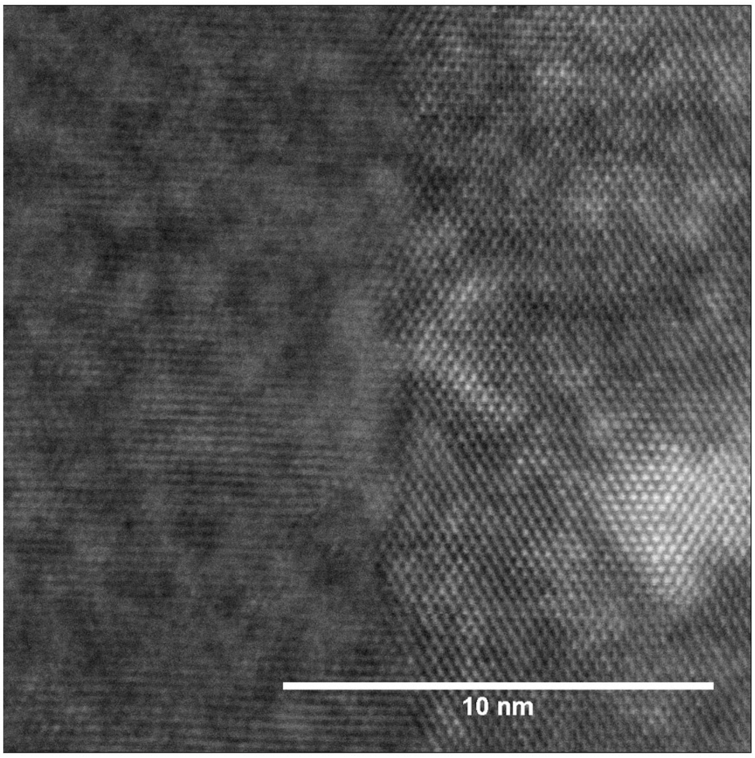

Fig. SI 2 HR-TEM of the TaW(C) film

*Corresponding author: e-mail stefan.fritze@kemi.uu.se, phone +46 18 4713724, fax +46 18 513548
